# Obesity and Cardio-Metabolic Risk Factors in an Urban and Rural Population in the Ashanti Region-Ghana: A Comparative Cross-Sectional Study

**DOI:** 10.1371/journal.pone.0129494

**Published:** 2015-06-05

**Authors:** Christian Obirikorang, Derick Nii Mensah Osakunor, Enoch Odame Anto, Samuel Opoku Amponsah, Opei Kwafo Adarkwa

**Affiliations:** 1 Department of Molecular Medicine, School of Medical Sciences, College of Health Sciences, Kwame Nkrumah University of Science and Technology, Kumasi, Ghana; 2 Department of Medical Laboratory Technology, Faculty of Allied Health Sciences, College of Health Sciences, Kwame Nkrumah University of Science and Technology, Kumasi, Ghana; 3 Department of Obstetrics and Gynaecology, Komfo Anokye Teaching Hospital, Kumasi, Ghana; University of Arkansas for Medical Sciences, UNITED STATES

## Abstract

There is a surge in chronic diseases in the developing world, driven by a high prevalence of cardio-metabolic risk factors. This study described differences in prevalence of obesity and cardio-metabolic risk factors between urban and rural settlements in the Ashanti Region of Ghana. This comparative cross-sectional study included 672 participants (median age 50 years), of which 312 were from Kumasi (urban) and 360 from Jachie-Pramso (rural). Demographic, anthropometric and other cardio-metabolic risk factors were gathered and venous blood samples were drawn for biochemical assays. Results suggested significant differences in diastolic blood pressure (80.0 mmHg vs 79.5 mmHg; p = 0.0078), and fasting blood sugar (5.0 mmo/l vs 4.5 mmol/l; p < 0.0001) between the two groups. Further differences in anthropometric measures suggested greater adiposity amongst participants in the urban area. Participants in the urban area were more likely than rural participants, to have high total cholesterol and LDL-c (p < 0.0001 respectively). Risk factors including BMI ≥ 25 (p < 0.0001), BMI ≥ 30 (p < 0.0001), high waist circumference (p < 0.0001), high waist-to-height ratio (p < 0.0001) and alcohol consumption (p = 0.0186) were more prevalent amongst participants in the urban area. Markers of adiposity were higher amongst females than males in both areas (p < 0.05). In the urban area, hypertension, diabetes and lifestyle risk factors were more prevalent amongst males than females. Differences in risk factors by urban / rural residence remained significant after adjusting for gender and age. Obesity and cardio-metabolic risk factors are more prevalent amongst urban settlers, highlighting an urgent need to avert the rise of diet and lifestyle-related chronic diseases.

## Introduction

The increase in non-communicable and chronic diseases in the developing world is an issue of major concern and can lead to reduced quality of life and premature deaths [1]. The prevalence of diabetes amongst adults in Africa as at 2010 was 3.8% and this is estimated to increase to 4.6% by the year 2030 [2]. Furthermore, the 1.2 million death toll attributed to cardiovascular diseases in Africa is expected to double by the year 2030 [3].

Prevalence of cardiovascular disease and diabetes is largely driven by cardio-metabolic risk factors such as smoking, lack of physical activity, low fruit and vegetable intake, high fat and salt intake, hypertension, abdominal obesity, dyslipidaemia, and excess alcohol intake [4]. The upward trend of cardio-metabolic diseases in Africa is likely to be due to an increase in these identifiable risk factors amongst the populace.

Overweight and obesity (abnormal or excessive fat accumulation that presents a risk to health) are strong risk factors for cardio-metabolic and other chronic medical conditions [5–7], including hypertension [8] and dyslipidaemia [9, 10]. The Body Mass Index (BMI), is the crude method of measure of overweight and obesity [11] but there are other popular but simple measures of obesity employed in recent times, which include the waist-to hip ratio (WHR), waist-to-height ratio (WHtR) and the percentage body fat (BF%) [5, 12], whose validity has been supported [13, 14].

Estimates from the World Health Organization (WHO) in 2008 showed that more than 1.4 billion adults, 20 years and older, were overweight with an overall 10% of the world’s population being obese [11]. There are reports on the prevalence of obesity across countries in and outside Africa [15, 16] and in Ghana [17–19], suggesting that obesity is increasing in prevalence [20]. It is reported that the prevalence of overweight and obesity in developing countries like Ghana is more rapid than in the developed world [21] and that by 2025 three-quarters of the obese population worldwide will be in non-industrialized countries [22].

The prevalence of overweight and obesity has risen steadily in Ghana over the years [17] and is reportedly more common in the capital city, Accra where urbanization is highest [18, 23]. Ghana has 10 administrative regions and data on other regions is limited. The Ashanti Region is the third largest with rapid urbanization, hence calls for urgent attention.

In Ghana, there is little available data on the prevalence of cardio-metabolic risk factors and its variations across compared populations. The current study therefore sought to describe the differences in prevalence of obesity and cardio-metabolic risk factors in Ghanaian adults drawn from urban and rural settlements.

## Materials and Methods

### Study Design and Site

This comparative cross-sectional study was conducted in two locations, Kumasi and Jachie-Pramso, both located in the Ashanti Region of Ghana, from January to July 2013.

Kumasi was classified as urban and Jachie-Pramso was classified as rural, based on population, settlement, location and way of life of the people. Kumasi is amongst the largest metropolitan areas of Ghana and the second most populous [24]. It is the commercial, industrial and cultural capital of the Ashanti Region. Jachie-Pramso is a village with a clustered human settlement, located in the Bosomtwe district [24] of the Ashanti region.

### Study Population

A total of 672 participants, consisting of 312 urban and 360 rural settlers were recruited via simple random sampling technique. Kumasi consists of 50 towns, of which 10 neighbourhoods were selected at random and 16 households selected at random from each neighbourhood. We selected at least one (1) eligible participant from each household. Due to the relatively smaller size of Jachie-Pramso, we visited about 80 households at random and eligible participants in each household were recruited. The sample size from each community was deemed representative of the apparently healthy population in both communities.

An interview-based questionnaire was designed in the English language but translated into the local dialects (mainly Twi), where appropriate. The questionnaire gathered information on socio-demographic and cardio-metabolic risk factors. Sample questionnaires were pre-tested before commencement of the study.

Participants with prior history of hypertension, diabetes or coronary heart disease were excluded. Documented evidence of treatment related to such conditions in previously diagnosed persons, also constituted exclusion from the study. We excluded pregnant and lactating women as well as physically and mentally disabled individuals.

### Biochemical Assays

After observing an overnight fast (12–14 hours), about 6 ml venous blood sample was taken from each participant into fluoride oxalate and serum separator tubes (about 3 ml each). Blood samples were centrifuged within 2 hours at 500 g for 15 min.

Plasma glucose levels were determined using the glucose oxidase method. Serum lipid levels; total cholesterol (TC), triglycerides (TG) and HDL-cholesterol (HDL-c) were determined by enzymatic methods on the Selectra Pro “S” System (Elitech Clinical Systems Elitech Group). LDL-cholesterol (LDL-c) was calculated using the Friedwald’s formula [25].

Dyslipidemia was defined as TC ≥ 5.20 mmol/l, HDL-c < 1.04 mmol/l, LDL-c ≥ 3.38 mmol/l TG ≥ 1.71 mmol/l and TC/HDL-c ratio ≥ 5 as per the United States National Cholesterol Education Program, Adult Treatment Panel (NCEP-ATP) III guidelines [26]. Diabetes was defined as fasting blood sugar (FBS) of ≥ 7.0 mmol/l [27].

### Anthropometric measurements

Body weight (to the nearest 0.1 Kg) in light clothing was measured with a mechanical scale (Hospibrand ZT-120, England). Height (to the nearest 0.1 cm) without shoes was measured with a commercial stadiometer (SECA, Germany).

The body mass index (BMI) was calculated using the formula; weight (Kg) / height (m2). Waist circumference (WC) (to the nearest 0.1 cm) was measured using a measuring tape (Gay Mills, WI), midpoint between the last palpable rib and the suprailiac crest, with the subjects standing and breathing normally [28]. The hip circumference (HC) was measured at the outermost points of the greater trochanters [29]. The waist and hip circumferences were measured with the tape parallel to the floor. The waist-to-hip ratio (WHR) was calculated using the formula; WC (cm) / HC (cm). The waist-to-height ratio (WHtR) was calculated using the formula; WC (cm) / height (cm).

Overall obesity was defined as a BMI of ≥ 30 Kg/m2 and obesity class I, II and III as a BMI of 30–34.9, 35–39.9 and ≥ 40 Kg/m2 respectively [30]. Abdominal obesity was defined as a waist circumference of > 94 cm in men or > 80 cm in women [30]. The cut off point for waist-to-hip ratio was ≥ 0.90 cm for men and ≥ 0.85 cm for women [30] and that of WHtR for both sexes was ≥ 0.50 [31]. Percentage body fat (BF%) was estimated using the following formula [32]; *Adult body fat*% = (1.20 × *BMI*) +(0.23 × *Age*)-(10.8 × *sex*) -5.4. The cut-offs for BF% for men and women were ≥ 25% and ≥ 30%, respectively [33].

### Blood pressure

Trained personnel measured the blood pressure of participants in accordance with recommendations of the American Heart Association [34]. The measurements were repeated after a minimum of 20 minutes [28] and mean values of duplicate measurements were recorded as the blood pressure. Hypertension was defined as a systolic blood pressure of ≥ 140 mmHg and/or diastolic blood pressure was ≥ 90 mmHg on two occasions, after initial screening (also with newly detected cases) [28, 35].

### Occupation

Occupational data gathered included a vast majority of jobs and these were classified as formal and informal based on set criteria. “Formal” jobs were defined as that which encompasses all jobs with normal hours, regular wages and are recognized as income sources on which income taxes must be paid. Any such contrary to this was classified as “informal”.

### Type of family

Participants who lived alone in their homes were categorized as “individual”, whiles those who lived with their immediate nuclear family (mother, father and children) were categorized as “nuclear” and those who lived with other family dependents (uncles, grandparents and in-laws) were categorized as living with “extended” families.

### Statistical methods

Data was analysed using MedCalc, version 12.7 and GraphPad Prism version 5.0. Continuous variables were expressed as medians and interquartile ranges (IQR). Categorical variables were expressed as frequencies and proportions. As continuous variables were not normally distributed, a non-parametric tests, the Mann-Whitney test (Rank sum test) was used to compare medians. The Chi-square or Fisher’s test was used to compare categorical variables as appropriate. Multivariate logistic regression was used to calculate the odds ratio describing the association of cardio-metabolic risk factors with urban residence, adjusted for age and gender. Spearman’s rho (Rank) correlation was used to determine the associations between various variables within each population group. Findings were considered statistically significant when p < 0.05.

### Ethical consideration

The study was approved by the Committee on Human Research, Publication and Ethics of the School of Medical Sciences (SMS), Kwame Nkrumah University of Science and Technology (KNUST); **Ref-CHRPE/RC/093/13. P**articipation was voluntary and written informed consent was obtained from each participant.

## Results

Table 1 shows the socio-demographic characteristics of the study population. Median age was 50 years and there was no significant difference in age distributions between participants in the urban and rural areas. The majority (62.5%) of families lived in the nuclear family system with a majority average income of GHS ≤ 500 (60.7%). There were significant differences between urban and rural participants with respect to gender distribution (p = 0.0004), marital status (p < 0.0001), employment (p < 0.0001), education (p < 0.0001), average income (p < 0.0001), type of family (p < 0.0001), and alcohol intake (p = 0.0186).

**Table 1:** Socio-demographic characteristics of the study population.

Variables	Total (n = 672)	Rural (n = 360)	Urban (n = 312)	*p*-value
**Age *(years)***				
Median *(IQR)*	50.0 (39.0–58.0)	50.0 (38.0–59.0)	49.0 (40.0–58.0)	0.8191
20–30 *n (%)*	66 (9.8)	36 (10.0)	30 (9.6)	
31–40 *n (%)*	130 (19.3)	70 (19.4)	60 (19.2)	
41–50 *n (%)*	202 (30.1)	106 (29.4)	96 (30.8)	
51–60 *n (%)*	217 (32.3)	115 (31.9)	102 (32.7)	
>60 *n (%)*	57 (8.5)	33 (9.2)	24 (7.7)	0.9648
**Gender *n (%)***				
Male	312 (46.4)	144 (40.0)	168 (53.8)	
Female	360 (53.6)	216 (60.0)	144 (46.2)	**0.0004**
**Marital status *n (%)***				
Single	75 (11.2)	57 (15.8)	18 (5.8)	
Married	531 (79.0)	249 (69.2)	282 (90.4)	
Divorced	42 (6.2)	30 (8.3)	12 (3.8)	
Widowed	24 (3.6)	24 (6.7)	0 (0.0)	**< 0.0001**
**Occupation *n (%)***				
Formal	261 (38.8)	105 (29.2)	156 (50.0)	
Informal	402 (59.8)	246 (68.3)	156 (50.0)	
Unemployed	9 (1.3)	9 (2.5)	0 (0.0)	**< 0.0001**
**Level of education *n (%)***				
Illiterate	36 (5.4)	36 (10.0)	0 (0.0)	
Basic	246 (36.6)	174 (48.3)	72 (23.1)	
SHS/Technical	177 (26.3)	69 (19.2)	108 (34.6)	
Tertiary	213 (31.7))	81 (22.5)	132 (42.3)	**< 0.0001**
**Average income (GHS) *n (%)***				
≤ 500	408 (60.7)	258 (71.7)	150 (48.1)	
501–999	120 (17.9)	54 (15.0)	66 (21.2)	
≥ 1000	144 (21.4)	45 (12.5)	96 (30.8)	**< 0.0001**
**Size of family *n (%)***				
Individual	57 (8.5)	27 (7.5)	30 (9.6)	
Nuclear	420 (62.5)	180 (50.0)	240 (76.9)	
Extended	195 (29.0)	153 (42.5)	42 (13.5)	**< 0.0001**
**Smoking *n (%)***				
No	642 (95.5)	348 (96.7)	294 (94.2)	
Yes	30 (4.5)	12 (3.3)	18 (5.8)	0.1377
**Alcohol intake *n (%)***				
No	543 (80.8)	303 (84.2)	240 (76.9)	
Yes	129 (19.2)	57 (15.8)	72 (23.1)	**0.0186**
**Exercise *n (%)***				
No	255 (37.9)	147 (40.8)	108 (34.6)	
Yes	417 (62.1)	213 (59.2)	204 (65.4)	0.1109

Data is presented as median (IQR); Mann-Whitney test or n (%); Chi-square or Fisher’s test. p < 0.05 was considered significant for rural vs urban. n: number, IQR: Interquartile range. GHS: Ghana Cedi.

As seen in Table 2, participants in the urban area had significantly higher measures of all selected anthropometric variables (p < 0.05), except for SBP (p > 0.05).

**Table 2:** Blood pressure and anthropometric variables of the study population.

Variables	Total (n = 672) *median (IQR*	Rural (n = 360) *median (IQR*	Urban (n = 312) *median (IQR*	*p*-value
**SBP** *(mmHg)*	133 (120–143)	133.5 (119–145)	133.0 (121–141)	0.8464
**DBP** *(mmHg)*	80.0 (75.0–86.0)	79.5 (72.0–87.0)	80.0 (77.0–85.0)	**0.0078**
**FBS** *(mmol/l)*	4.70 (4.3–5.2)	4.5 (4.1–5.0)	5.0 (4.5–5.4)	**< 0.0001**
**Weight** *(Kg)*	77.6 (66.0–85.4)	68.3 (56.5–79.8)	82.1 (75.9–88.3)	**< 0.0001**
**Height** *(m)*	1.65 (1.6–1.7)	1.64 (1.6–1.7)	1.67 (1.6–1.7)	**0.0001**
**BMI** *(Kg/m* ^*2*^ *)*	26.8 (23.9–31.6)	25.3 (20.4–29.8)	29.9 (25.6–32.6)	**< 0.0001**
**Waist circumference** *(cm)*	93.0 (85.5–98.0)	89.0 (78.0–95.0)	95.0 (91.0 (98.5)	**< 0.0001**
**Hip Circumference** *(cm)*	103 (96.5–112.0)	99.0 (90.5–109.0)	109.5 (101.5–114.0)	**< 0.0001**
**Waist to Hip Ratio (WHR)**	0.88 (0.84–0.92)	0.88 (0.84–0.92)	0.89 (0.85–0.93)	**0.0485**
**Waist to Height Ratio (WHtR)**	0.55 (0.51–0.60)	0.53 (0.47–0.59)	0.58 (0.53–0.62)t	**< 0.0001**
**Percentage Body Fat (BF%)**	45.2 (39.3–49.2)	41.2 (35.9–47.3)	47.1 (44.1–49.8)	**< 0.0001**

Data is presented as median (IQR); compared using Mann-Whitney test. p < 0.05 was considered significant for rural vs urban. n: number. IQR: Interquartile range. SBP: Systolic blood pressure. DBP: Diastolic blood pressure. FBS: Fasting blood sugar. BMI: Body mass index.

Participants in the urban area had significantly higher levels of serum cholesterol and LDL-c than that observed amongst participants in the rural area (p < 0.0001 respectively). This was however not true for serum triglyceride levels, as that of the urban participants was lower than that observed in rural participants (p < 0.0001). [Table 3]

**Table 3:** Serum lipid levels of the study population.

Variable	Total (n = 672) *median (IQR*	Rural (n = 360) *median (IQR*	Urban (n = 312) *median (IQR*	*p*-value
**Total Cholesterol *(*** *mmol/l)*	4.90 (4.55–5.40)	4.80 (4.55–5.20)	5.00 (4.65–5.50)	**< 0.0001**
**Triglycerides *(*** *mmol/l)*	1.30 (1.00–1.60)	1.35 (1.10–1.70)	1.20 (0.80–1.40)	**< 0.0001**
**HDL-c *(*** *mmol/l)*	1.00 (0.80–1.20)	1.00 (0.80–1.20)	1.00 (0.80–1.20)	0.8995
**LDL-c *(*** *mmol/l)*	3.40 (2.90–3.65)	3.10 (2.70–3.60)	3.40 (3.05–3.80)	**< 0.0001**
**Coronary risk**	4.9 (4.09–5.89)	4.89 (4.00–5.75)	4.95 (4.14–6.01)	0.0947

Data is presented as median (IQR); compared using Mann-Whitney test. p < 0.05 was considered significant for rural vs urban. n: number. IQR: Interquartile range. HDL-c: High Density Lipoprotein cholesterol. LDL-c: Low Density Lipoprotein Cholesterol. Coronary Risk: Total Cholesterol/HDL-c.

**In** Table 4, we compare selected cardio-metabolic risk factors by settlement (rural or urban). Participants in the urban area were more likely than rural residents, to have a high total cholesterol and LDL-c (p < 0.0001 respectively). There were more participants in the urban area with BMI ≥ 25 (p < 0.0001) and BMI ≥ 30 (p < 0.0001), high WC (p < 0.0001), high WHtR (p < 0.0001) and who consumed alcohol (p = 0.0186) than there was in the rural area, suggesting differences in adiposity between the two areas.

**Table 4:** Cardio-metabolic risk factors amongst study population stratified by type of community.

Risk factors	Total (n = 672) *n (%)*	Rural (n = 360) *n (%)*	Urban (n = 312) *n (%)*	*p*-value
**Hypertension** *(≥ 140/90 mmHg)*	234 (34.8)	132 (36.7)	102 (32.7)	0.2921
**Type II diabetes** *(FBS > 7*.*0 mmol/l)*	51 (7.6)	21 (5.8)	30 (9.6)	0.0792
**Total cholesterol** *(≥ 5*.*20 mmo/l)*	231 (34.4)	99 (27.5)	132 (42.3)	**< 0.0001**
**LDL-c** *(≥ 3*.*38 mmol/l)*	339 (50.4)	147 (40.8)	192 (61.5)	**< 0.0001**
**HDL-c** *(< 1*.*04 mmo/l)*	402 (59.8)	216 (60.0)	186 (59.6)	0.9372
**Triglycerides** *(≥ 1*.*71mmol/l)*	105 (15.6)	75 (20.8)	30 (9.6)	**< 0.0001**
**BMI** *(≥ 25 Kg/m* ^*2*^ *)*	471 (70.1)	201 (55.8)	270 (86.5)	**< 0.0001**
**Overall obesity** *(≥ 30 Kg/m* ^*2*^ *)*	243 (36.2)	87 (24.2)	156 (50.0)	**< 0.0001**
**WC** *(men > 94 cm; women > 80 cm)*	381 (56.7)	177 (49.2)	204 (65.4)	**< 0.0001**
**WHR** *(men ≥ 0*.*90; women ≥ 0*.*85)*	396 (58.9)	210 (58.3)	186 (59.6)	0.7536
**WHtR**, *(≥ 0*.*50)*	546 (81.2)	246 (68.3)	300 (96.2)	**< 0.0001**
**BF%** *(men ≥ 25%; women ≥ 30%)*	669 (99.6)	357 (99.2)	312 (100.0)	0.2527
**Physical inactivity**	255 (37.9)	147 (40.8)	108 (34.6)	0.1109
**Smoking habit**	30 (4.5)	12 (3.3)	18 (5.8)	0.1377
**Alcohol intake**	129 (19.2)	57 (15.8)	72 (23.1)	**0.0186**

Data is presented as n (%); compared using Fischer’s test. p < 0.05 was considered significant for rural vs urban. BMI: Body mass index. WC: Waist Circumference. WHR: Waist-to hip Ratio. WHtR: Waist-to height Ratio. BF%: Percentage body fat. HDL-c: High Density Lipoprotein cholesterol. LDL-c: Low Density Lipoprotein Cholesterol.

After adjusting for gender and age, differences in cardio-metabolic risk factors between participants in the two areas remained significant, with participants in the urban area more likely to have cardio-metabolic risk factors. [Table 5]

**Table 5:** Odds Ratios associated with selected cardio-metabolic factors amongst the urban population (reference: rural population).

Risk factors	Crude OR (95% CI)	*p*-value	Adj. OR-gender (95% CI)	*p-*value	Adj. OR- gender and age (95% CI)	*p*-value
**Total cholesterol** *(≥ 5*.*20 mmo/l)*	1.9333 (1.40–2.67)	0.0001	1.9309 (1.39–2.67)	0.0001	1.8923 (1.36–2.62)	0.0001
**LDL-c** *(≥ 3*.*38 mmol/l)*	2.3184 (1.70–3.16)	<0.0001	2.1443 (1.56–2.94)	<0.0001	2.1554 (1.56–2.97)	<0.0001
**Triglycerides** *(≥ 1*.*71mmol/l)*	0.4043 (0.26–0.64)	0.0001	0.4151 (0.26–0.66)	0.0002	0.4058 (0.25–0.65)	0.0001
**BMI** *(≥ 25 Kg/m* ^*2*^ *)*	5.0853 (3.46–7.48)	<0.0001	6.4125 (4.26–9.66)	<0.0001	6.1730 (4.07–9.37)	<0.0001
**Overall obesity** *(≥ 30 Kg/m* ^*2*^ *)*	3.1379 (2.26–4.35)	<0.0001	7.1574 (4.62–11.08)	<0.0001	6.7235 (4.35–10.39)	<0.0001
**WC** *(men > 94cm; women > 80 cm)*	1.9529 (1.46–2.67)	<0.0001	4.6587 (3.00–7.23)	<0.0001	4.3237 (2.76–6.76)	<0.0001
**WHtR**, *(≥ 0*.*50)*	11.5854 (6.24–21.50)	<0.0001	13.4726 (7.18–25.28)	<0.0001	12.7659 (6.76–24.11)	<0.0001
**Alcohol intake**	1.5947 (1.08–2.35)	0.0180	1.3866 (0.93–2.07)	0.1078	1.6292 (1.08–2.46)	0.0198

OR: Odds ratio. Adj: adjusted. Compared using Multivariate logistic regression. p < 0.05 was considered significant.

The selected cardio-metabolic risk factors are further stratified by gender in Table 6, Male participants in the urban area were significantly more likely to have cardio-metabolic risk factors (total cholesterol, LDL-c, HDL-c, BMI ≥ 25, WC, WHtR) than male participants in the rural area. Similarly, there were significantly more female participants in the urban area with high cholesterol, BMI ≥ 25, BMI ≥ 30, high waist circumference and high WHtR than there was in the rural area.

**Table 6:** Cardio-metabolic risk factors amongst study population stratified by gender and type of community.

Risk factors	Male (n = 312)			Female (n = 360)		
	Rural (n = 144) *n (%)*	Urban (n = 168) *n (%)*	*p*-value	Rural (n = 216) *n (%)*	Urban (n = 144) *n (%)*	*p*-value
**Hypertension** *(≥ 140/90 mmHg)*	72 (49.9)****	72 (42.9)	0.2616	60 (27.8)	30 (20.8)†††	0.1681
**Type II diabetes** *(FBS > 7*.*0 mmol/l)*	12 (8.3)	24 (14.3)	0.1398	9 (4.2)	6 (4.2) ††	0.7886
**Total cholesterol** *(≥ 5*.*20 mmo/l)*	39 (27.1)	72 (42.9)	**0.0053**	60 (27.8)	60 (41.7)	**0.0086**
**LDL-c** *(≥ 3*.*38 mmol/l)*	66 (45.8)	126 (75.0)	**<0.0001**	81 (37.5)	66 (45.8) ††††	0.1442
**HDL-c** *(< 1*.*04 mmo/l)*	69 (47.9)***	102 (60.7)	**0.0053**	147 (68.1)	84 (58.3)	0.0738
**Triglycerides** *(≥ 1*.*71mmol/l)*	24 (16.7)	18 (10.7)	0.1672	51 (23.6)	12 (8.3)	**0.0003**
**BMI** *(≥ 25 Kg/m* ^*2*^ *)*	69 (47.9) *	126 (75.0)	**<0.0001**	132 (61.1)	144 (100.0) ††††	**<0.0001**
**Overall obesity** *(≥ 30 Kg/m* ^*2*^ *)*	15 (10.4)****	30 (17.9)	0.0859	72 (33.3)	126 (87.5) ††††	**<0.0001**
**WC** *(men > 94cm; women > 80cm)*	30 (20.8)****	60 (35.7)	**0.0056**	147 (68.1)	144 (100.0) ††††	**<0.0001**
**WHR** *(men ≥ 0*.*90; women ≥ 0*.*85)*	75 (52.1)	96 (57.1)	0.4410	135 (62.5)	90 (62.5)	1.0000
**WHtR**, *(≥ 0*.*50)*	87 (60.4)*	156 (92.9)	**<0.0001**	159 (73.6)	144 (100.0) ††	**<0.0001**
**BF%** *(men ≥ 25%; women ≥ 30%)*	144 (100.0)	168 (100.0)	1.0000	213 (98.6)	144 (100.0)	0.4029
**Physical inactivity**	42 (29.2)***	54 (32.1)	0.6670	105 (48.6)	54 (37.5)	**0.0489**
**Smoking habit**	9 (6.2)*	18 (10.7)	0.2265	3 (1.4)	0 (0.0) †††	0.4029
**Alcohol intake**	42 (29.2)****	48 (28.6)	0.9930	15 (6.9)	24 (16.7) †	**0.0058**

Data is presented as n (%); compared using Fisher’s test. p < 0.05 was considered significant for rural vs urban in each gender category.

* Indicate significance for comparison between rural males and rural females.

† Indicate significance for comparison between urban males and urban females.

*/† Significant at the p < 0.05 level.

**/†† Significant at the p < 0.01 level.

***/††† Significant at the p < 0.001 level.

****/†††† Significant at the p < 0.0001 level. n: number BMI: Body mass index. WC: Waist Circumference. WHR: Waist-to hip Ratio. WHtR: Waist-to height Ratio. BF%: Percentage body fat. HDL-c: High Density Lipoprotein cholesterol. LDL-c: Low Density Lipoprotein Cholesterol

In the rural area, low HDL-c (p < 0.001), BMI ≥ 25 (p < 0.05), BMI ≥ 30 (p < 0.0001), high WC (p < 0.0001) high WHtR (p < 0.05) and physical inactivity (p < 0.001) were more prevalent amongst females than their corresponding males. [Table 6]

Of those in the urban area, there were more females than males with a BMI ≥ 25 (p < 0.0001), overall obesity (p < 0.0001), high WC (p < 0.0001) and high WHtR (p < 0.01). There were however more males than females with hypertension (p < 0.001), type II diabetes (p < 0.01), LDL-c (p < 0.0001), a smoking habit (p < 0.001) and who consumed alcohol (p < 0.05). [Table 6]

For participants in both the urban and rural areas, there was a significant positive correlation between SBP and DBP, between blood pressure (SBP and DBP) and BF% and so was BMI with WC, WHR, BF%, cholesterol and LDL-c. Similarly, waist circumference correlated positively with WHR, WHtR, BF%, cholesterol and LDL-c and so was cholesterol with triglycerides and LDL-c, in both areas. WHR showed a significant positive association with BF% in both areas but with WHtR only in the rural community and with cholesterol and LDL-c in the urban community. A positive association was observed between FBS and blood pressure (SBP and DBP) in the urban area but with only DBP in the rural area. LDL-c showed a significant positive association with triglycerides and BF%, amongst participants in the urban area. [Table 7]

**Table 7:** Spearman’s rho correlation coefficients between selected cardio-metabolic variables for rural (Lower Left-Hand Side) and urban (Upper Right-Hand Side).

Variables	SBP	DBP	FBS	BMI	WC	WHR	WHtR	BF%	TCHL	HDL-c	LDL-c	TG
**SBP**		**.697** **	**.289** *	-.086	.043	.198	-.042	**.340** *	.014	-.178	.152	.003
**DBP**	**.759** **		**.443** **	-.075	-.017	.010	-.115	**.297** *	.057	-.028	.082	.075
**FBS**	.133	**.256** **		-.152	-.114	.078	-.170	.106	.066	-.072	.112	.071
**BMI**	.016	.116	.078		**.731** **	-.158	**.864** **	**.344** *	**.293** *	-.040	**.250** *	.093
**WC**	.035	.106	.150	**.755** **		**.338** *	**.897** **	**.381** **	**.203** *	-.230	**.277** *	.115
**WHR**	.070	-.002	.089	**.190** *	**.663** **		.118	**.382** **	**.219** *	**-.492** **	**.210** *	-.132
**WHtR**	.024	.065	.079	**.829** **	**.935** **	**.603** **		.218	-.079	-.154	**.278** *	.106
**BF%**	.200*	**.184** *	**.199** *	**.645** **	**.563** **	**.321** **	**.521** **		-.063	-.263	.077	.099
**TCHL**	.008	.127	.144	**.212** *	**.274** **	.089	**.280** **	.169		.171	**.818** **	**.541** **
**HDL**	.008	.019	-.095	-.100	-.161	**-.184** *	-.155	-.035	.106		-.194	.061
**LDL**	-.013	.105	**.206** *	**.263** **	**.322** **	.124	**.321** **	.**228** *	**.826** **	**-.257** **		**.286** *
**TG**	.075	.010	-.049	-.122	-.054	.080	-.011	-.138	**.224** *	-.062	-.068	

** Correlation is significant at the 0.01 level (2-tailed).

* Correlation is significant at the 0.05 level (2-tailed). SBP: Systolic Blood Pressure. DBP: Diastolic blood Pressure. FBS: Fasting Blood Sugar. WC: Waist circumference. WHR: Waist-to-hip ratio. WHtR: Waist-to-height ratio. BF%: percentage Body Fat. TCHL: Total Cholesterol. HDL-c: High Density Lipoprotein cholesterol. LDL-c: Low Density Lipoprotein Cholesterol. TG: triglycerides.

## Discussion

As urbanization continues to reach many communities in Ghana, there is a possible risk of a change in lifestyle. This may result in changes cardio-metabolic risk factors, which may reduce the quality of life and increase the rates of premature deaths. In this study, we described the differences in prevalence of obesity and cardio-metabolic risk factors, in study participants residing in urban and rural settlements. Results showed that indices of obesity (BMI, WC, HC, WHR, WHtR, and BF%) were higher in urban residents. Similarly, other cardio-metabolic markers (DBP, FBS, total cholesterol and LDL-c) and unhealthy lifestyle practices like alcohol consumption were higher amongst urban residents. Cardio-metabolic risk factors were observed to be more prevalent in female participants, as compared to males, after adjusting for age.

Participants were observed to have high rates of literacy and employment; rural residents were more likely than urban residents to work in the informal sector. The informal sector usually comprises of jobs mostly classified as “non-white collar” and involves activity that is more vigorous and requires less number of years of education, compared to jobs in the formal sector. The high rate of informal sector employment amongst the rural participants may therefore be due to the low level of education in this area. Occupational physical activity may contribute to the lower prevalence of cardio-metabolic risk factors in rural, compared to urban residents, as observed in this study. A study conducted in 2007 reported that residency per se does not contribute solely to the differences in cardio-metabolic risk factors observed between urban and rural areas in Guatemalan adults; rather, occupation is a more important factor [36]. Gregory and colleagues reported a similarly lower prevalence of cardio-metabolic risk factors amongst people with jobs that involved less sedentary lifestyles (rural) compared to those in the urban areas. Therefore, residency, education and occupation interact and may have implications on lifestyle factors, which affect the observed rate of cardio-metabolic risk factors amongst a population.

Many similar studies have shown strong evidence of associations between increasing urbanization and cardio-metabolic risk factors like high BMI, diabetes mellitus, or hypertension [3, 37–40]. Theories propose that as areas adopt urban characteristics, energy consumption and expenditure patterns change through a rise in energy-rich diets and sedentary lifestyles [41, 42]. These changes in effect result in an increase in adiposity and thus an increase in cardio-metabolic risks. A cross-sectional study in Cameroon similarly reported that urban living was associated with a higher fasting blood sugar, a higher BMI, and a higher blood pressure compared to rural residents with less than 2 years of exposure to urban environments [43]. The present study however could not determine the effect of time on the change in lifestyle and cardio-metabolic risk factors. The findings we report here are still important for public health as they suggest that people living in urban areas have increased risk factors to cardio-metabolic diseases.

It is however noteworthy that along with the observed differences in prevalence of cardio-metabolic risk factors, we recorded more females in the urban community and more males in the rural community. Furthermore, cardio-metabolic risk factors were observed to be more prevalent in female participants, Research in Ghana has shown that cardio-metabolic risk factors are more prevalent amongst women than in men [18, 19]. Amoah and colleagues found that at all ages, more females (32.9%) than males (12.0%) had BMI’s within the 4th quartile (> or = 27.3 Kg/m2) [18]. Gregory *et al*., in a similar study showed that sedentary lifestyle is more common amongst women than in men [36] and this may have contributed to the observed higher prevalence of cardio-metabolic risk factors in the urban area. High class or urban females have also been shown to have higher BMI than females in lower class suburbs [18]. When stratified by gender, the differences in cardio-metabolic risk factors were seen in each strata. When adjusted for sex, the differences between urban and rural participants remained statistically significant. This suggests that the urban / rural differences observed are not driven by the prevalence of women in these settings, but by the interaction of both gender and setting.

Overweight and obesity are important risk factors for cardio-metabolic and diverse chronic medical conditions [5–7], including hypertension [8] and dyslipidaemia [9, 10]. This is supported by our observed positive correlation between anthropometric variables, serum lipids (total cholesterol and LDL-c) and blood pressure in both areas studied.

The current study is however limited by its cross-sectional nature. As such, we were unable to determine the changes in lifestyle that occur over time in both urban and rural areas. The current study also did not take into account residential history of participants before recruitment. Percentage body fat calculation using BMI has not been validated in the African population. Furthermore, Deurenberg *et al*., [32] showed that BF% in obese subjects (BMI > 30 Kg/m2) was slightly overestimated by the prediction formula used in the present study. This difference however became statistically significant only in obese subjects with a BMI ≥ 33 Kg/m2. In the present study however, median BMI for the total population, and for both areas were less than 30 Kg/m2 and the proportion of individuals with a BMI >30, was 36.2%. The addition of other anthropometric variables further improves findings from the present study.

## Conclusion

Obesity and cardio-metabolic risk factors are prevalent amongst adults in both urban and rural communities in Ghana. These risk factors are more prevalent in urban residents and in women., There is an urgent need to avert the rise of diet and lifestyle related chronic diseases in Ghana as it undergoes increasing urbanization. It would be prudent for health workers to incorporate measures of adiposity into routine vital checks to enhance evaluation of health status of individuals.
